# Improvement of long segment ribosomal PCR amplification for molecular identification of *Litylenchus crenatae mccannii* associated with beech leaf disease

**DOI:** 10.21307/jofnem-2020-016

**Published:** 2020-03-17

**Authors:** L.K. Carta, S. Li

**Affiliations:** 1 Mycology and Nematology Genetic Diversity and Biology Laboratory, USDA, ARS, Henry A. Wallace Beltsville Agricultural Research Center, Bldg. 010 A, Room 110, Beltsville, MD 20705-2350

**Keywords:** Long segment nematode PCR, Ribosomal DNA marker, Single nematode crude genomic DNA, Technical improvement

## Abstract

Generating DNA markers for microscopic plant parasitic nematodes can be especially difficult if only a few valuable, tiny specimens are available. Providing a reliable maximum amount of unambiguous genetic information from single nematodes is especially important when identifying damaging, regulated nematodes of importance to trade where a few nucleotide differences in diagnostic markers are significant. There are many possible reasons for difficulty amplifying unpurified nematode DNA for long range PCR followed by direct sequencing. Specimen age, proofreading errors and reagent compatibility during PCR are among those problems. While unsuccessful direct amplification of difficult samples may sometimes be overcome by cloning, a more expensive and time-consuming process. Therefore, long segment PCR of a large 3.5 kb segment of ribosomal DNA was optimized for individual difficult-to-amplify young *Litylenchus crenatae mccannii* (Anguinidae) nematodes by systematically testing thermostable polymerases, proofreading enzymes and buffers. The combination of thermostable DreamTaq™, proofreading *Pfu* polymerase, and PicoMaxx™ buffer provided the best results. These nematodes are the subject of surveys currently active at many sites in the northeastern United States. This new, optimized PCR protocol will be useful for diagnostic labs associated with the surveys.

Beech leaf disease (BLD) is an emerging tree disease in the Northeast for American beech (*Fagus grandifolia*) trees in Ohio, Pennsylvania, New York and Connecticut. It was discovered first in 2012 near Lake Erie in the Cleveland Metroparks, region of Ohio (Pogacnik and Macy, 2016). The leaves that host BLD have noticeable symptoms of banded dark green to chlorotic lesions between veins that increase in intensity and nematode numbers from summer to autumn. Many American beech trees with BLD may die eventually and this loss would devastate the beech-maple forest ecosystems of the eastern USA. The etiology was not understood (Ewing et al., 2019) until recently when a nematode was demonstrated to be necessary for experimental symptom production (Carta et al., 2020). As the disease spreads, accurate identification of nematodes in new symptomatic trees is more important than ever to delimit and potentially contain the spread of the disease and to identify the nematode in surveys outside the USA where related species may exist.

Molecular and morphological taxonomic identifications were conducted in our lab with the nematodes isolated from the lesions of the BLD leaves collected in Fall, 2017 from Perry, Lake County, Ohio, USA by an Ohio Department of Agriculture nursery inspector from ailing American beech trees *Fagus grandifolia* (Fall specimens). Their ribosomal DNA (rDNA) loci were amplified by PCR with the one primer set and an enhanced DNA polymerase system, and the resulting 3.5 kb (18 S, ITS and 28 S) rDNA amplicons were directly sequenced (Carta and Li, 2019). Morphological evaluation and an initial GenBank search revealed that this was an unknown species in the genus *Litylenchus*. Shortly after this discovery, a *Litylenchus* nematode from leaf galls of *Fagus crenata* in Japan was described as *Litylenchus crenatae* (Kanzaki et al., 2019), showing a few different DNA base pairs from the nematode population we sequenced. Based on these molecular, morphological and host range differences, a new subspecies *Litylenchus crenatae mccannii* was described (Carta et al., 2020).

Special handling of nematodes in PCR reactions is needed because of potential molecular marker variation within and between individual nematodes, plus their often tiny size and chemically resistant cuticle that may create unexpected challenges. Among more than 40 *Litylenchus* specimens from the samples collected from Ohio and Pennsylvania during the summer of 2018, 35 specimens with no PCR bands for the 3.5 kb target were observed, and the rest failed to yield a long enough target for downstream sequencing. This report describes a significant technical improvement beyond previous efforts (Carta and Li, 2018, 2019) to more reliably amplify the 3.5 kb long rDNA target and increase the PCR yield for the crude, unpurified DNA extracts of single nematodes by utilizing proofreading DNA polymerase in an optimized solution. This is important because it is impractical in a nematode diagnostic laboratory to efficiently produce very clean DNA with a kit from only one or a few specimens.

Commonly used *Taq* DNA polymerase lacks proofreading ability, which limits the length of the amplicon, usually up to 2.9 kb (Arezi et al., 2003; Tindall and Kunkel, 1988). It has been demonstrated that long template DNA could be amplified successfully by adding a thermal proofreading DNA polymerase with 3’ to 5’ exonuclease activity to a *Taq* PCR system (Barnes, 1994; Cheng et al., 1995). As a result, many PCR amplification systems have been developed and made commercially available by blending a *Taq* polymerase and a thermal proofreading DNA polymerase supplied with a specially optimized PCR buffer. Two *Taq*-based blend systems, TaKaRa Ex *Taq*® DNA Polymerase (a blend of TaKaRa *Taq*® DNA Polymerase and an unspecified proofreading DNA polymerase) and PicoMaxx™ High Fidelity PCR System (a blend of *Taq*2000™ DNA polymerase, cloned *Pfu* DNA polymerase and ArchaeMaxx® polymerase enhancing factor) were selected and tested in this study.

## Materials and methods

Live *Litylenchus* specimens were isolated from the banding lesions of American beech leaves with BLD as described in Table 1, after the leaves were dissected, and followed by water extraction. Some of the specimens were also imaged as vouchers for morphological and morphometrical analysis. The preparation of the crude and unpurified genomic DNA from a live single *Litylenchus* and the visualization, cleanup and direct DNA sequencing, including sequencing primers, of the PCR products were performed by using the procedures described in previous studies (Carta and Li, 2018, 2019).

**Table 1.** *Litylenchus crenatae* specimens from American beech trees (*Fagus grandifolia*) with BLD tested in this study.

Specimens	Locality	Part	Session
104H78, 104H81, 104H82, 104H83, 104H84, 104H85, 104H86, 104H87, 104H88, 104H89 and 104H90	Lake County, Ohio	Leaf	Fall (November, 2017)
104J54, 104J55, 104J56 and 104J57	Cuyahoga County, Ohio	Leaf	Summer (May, 2018)
104K17, 104K18, 104K19 and 104K20	The Holden Arboretum, Kirtland, Ohio	Leaf	Summer (August, 2018)
104K25, 104K26, 104K27, 104K28, 104K29, 104K30 and 104K31	Potter County, Pennsylvania	Leaf	Summer (August, 2018)
104K37, 104K38 and 104K39	Crawford County, Pennsylvania	Leaf	Summer (August, 2018)
104N95, 104N96 and 104N97	The Holden Arboretum, Kirtland, Ohio	Bud	Spring (March, 2019)

Either the 3.5 kb long segment or the 1.7 or 1.9 kb medium segment ribosomal amplifications by PCR with Dream*Taq*™ Hot Start DNA polymerase system (Dream*Taq*™, Thermo Fisher Scientific, Waltham, MA) were performed by using the procedures, including primer sets, 18S-CL-F3 and 28S-CL-R for the 3.5 kb ribosomal loci, 18S-CL-F3 and 18S-CL-R7 for the 18 S locus (1.7 kb) and ITS-CL-F2 and 28S-CL-R for the ITS-28S (D1D2D3) loci (1.9 kb) described in the previous study (Carta and Li, 2019) unless otherwise noted.

Assembling PCR buffer and parameterizing thermal cycling conditions in the following amplifications with different DNA polymerases were performed according to each respective manufacturers’ guidance. Treatment components are summarized in Table 2, and cycling conditions in Tables 3 and 4.

**Table 2.** PCR components and setup.

	Platinum™ *Taq* (10 units/μl)	*Taq*2000™ (5 units/μl)	Dream*Taq*™ (5 units/μl)	TaKaRa Ex *Taq*™ (5 units/μl) or combined with Dream*Taq*™ (5 units/μl)	PicoMaxx™ System (5 units/μl) or combined with Dream*Taq*™ (5 units/μl)	*pfu* DNA polymerase (2.5 units/μl) or combined with Dream*Taq*™ (5 units/μl)	Herculase® II Fusion DNA polymerase	Phusion™ High-Fidelity DNA Polymerase (2 units/μl)	*Pwo* DNA polymerase (5 units/μl) or combined with Dream*Taq*™ (5 units/μl)
Water (μl)									Mixture A: 7.6
Water (μl)	15.375	16	16.375/16.25	15.875 or 15.75	17.3 or 17.175	17.05/17.55 or 16.925/17.425	16	14.5	Mixture B: 9.875 (or 9.75)
10 or 5x proprietary buffer (μl)	2.5	2.5	2.5	2.5	2.5	2.5	5	5	Mixture A: 2.5
50 mM MgCl_2_ (μl)	1	0.25							
100 mM dNTP (25 mM each) (μl)				0.2	0.2	0.25			Mixture B: 0.4
10 mM dNTP (2.5 mM each) (μl)				2				0.5	
8 mM dNTP (2 mM each) (μl)	2.5	2.5	2.5						
10 μm Forward primer (μl)	0.75	0.75	0.75	1.25	1.25	1.25	0.625	1.25	Mixture B: 1.25
10 μm Reverse primer (μl)	0.75	0.75	0.75	1.25	1.25	1.25	0.625	1.25	Mixture B: 1.25
DMSO								0.25	
DNA template (μl)	2	2	2	2	2	2	2	2	Mixture B: 2
Proprietary DNA polymerase(s) (μl)	0.125	0.25	0.125/0.25	0.125 or plus Dream*Taq*™: 0.125	0.5 or plus Dream*Taq*™: 0.125	0.75/0.25 or plus Dream*Taq*™: 0.125	0.5	0.25	Mixture A: 0.125 or plus Dream*Taq*™: 0.125
Total reaction volume (μl)	25	25	25	25	25	25	25	25	12.5 each mixture

**Table 3.** PCR cycling conditions.

	Platinum™ *Taq*		*Taq*2000™		Dream*Taq*™		TaKaRa Ex *Taq*™ or combined with Dream*Taq*™		PicoMaxx™ System or combined with Dream*Taq*™	
1. Initial denaturation	95°C for 3 min	Sep 1: 1 cycle	95°C for 3 min	Step 1: 1 cycle	95°C for 3 min	Step 1: 1 cycle	98°C for 30 sec	Step 1: 1 cycle	95°C for 2 min	Step 1: 1 cycle
2. Denaturation	95°C for 30 sec	Step 2, 3 and 4: 36 cycles	95°C for 30 sec	Step 2, 3 and 4: 36 cycles	95°C for 30 sec	Step 2, 3 and 4: 36 cycles	98°C for 10 sec	Step 2 and 4: 36 cycles	95°C for 30 sec	Step 2, 3 and 4: 36 cycles
3. Annealing	50°C for 45 sec		50°C for 45 sec		50°C for 45 sec				55°C for 45 sec	
4. Extension	72°C for 3 min		72°C for 3 min		72°C for 3 min		68°C for 5 min		72°C for 5 min	
5. Final extension	72°C for 7 min	Step 5: 1 cycle	72°C for 7 min	Step 5: 1 cycle	72°C for 7 min	Step 5: 1 cycle	72°C for 7 min	Step 5: 1 cycle	72°C for 7 min	Step 5: 1 cycle

**Table 4.** PCR cycling conditions.

No. Step	*pfu* or combined with Dream*Taq*™		Herculase® II		Phusion™		*Pwo* or combined with Dream*Taq*™	
1. Initial denaturation	95°C for 2 min	Step 1: 1 cycle	95°C for 2 min	Step 1: 1 cycle	95°C for 2 min	Step 1: 1 cycle	95°C for 2 min	Step 1: 1 cycle
2. Denaturation	95°C for 30 sec	Step 2, 3 and 4: 36 cycles	95°C for 20 sec	Step 2, 3 and 4: 36 cycles	95°C for 20 sec	Step 2, 3 and 4: 36 cycles	95°C for 30 sec	Step 2, 3 and 4: 36 cycles
3. Annealling	55°C for 45 sec		55°C for 20 sec		55°C for 20 sec		57°C for 45 sec	
4. Extension	72°C for 5 min		72°C for 2 min 15 sec		72°C for 2 min 15 sec		72°C for 5 min	
5. Final extension	72°C for 7 min	Step 5: 1 cycle	72°C for 7 min	Step 5: 1 cycle	72°C for 7 min	Step 5: 1 cycle	72°C for 7 min	Step 5: 1 cycle

### Platinum™ *Taq* DNA polymerase

Each PCR amplification with Platinum™ *Taq* DNA Polymerase (Platinum™ *Taq*) (Thermo Fisher Scientific, Waltham, MA) was carried out in a 25 μl of mixture containing Platinum™ *Taq* (10 units/μl) 0.125 μl, 10X PCR Buffer Mg 2.5 μl, MgCl_2_ (50 mM) 1 μl, dNTP (2.0 mM each) 2.5 μl, Template DNA 2 μl, forward primer (10 μm) 0.75 μl and reverse primer (10 μm) 0.75 μl for either primer set 18S-CL-F3 and 28S-CL-R or ITS-CL-F2 and 28S-CL-R, and molecular biology grade water (Sigma-Aldrich, St Louis, MO) 15.375 μl. The thermal cycling program was one cycle of 95°C for 3 min; 36 cycles of 95°C for 30 sec, 50°C for 45 sec, 72°C for 3 min; and final extension at 72°C for 7 min.

### *Taq*2000™ DNA polymerase

Each PCR amplification with *Taq*2000™ DNA Polymerase (Agilent, Santa Clara, CA) was carried out in a 25 μl mixture containing *Taq*2000™ (5 units/μl) 0.25 μl, 10X PCR Buffer 2.5 μl, MgCl_2_ (50 mM) 0.25 μl, dNTP (2.0 mM each) 2.5 μl, Template DNA 2 μl, both forward primer (10 μm) 0.75 μl and reverse primer (10 μm) 0.75 μl for either primer set 18S-CL-F3 and 28S-CL-R or ITS-CL-F2 and 28S-CL-R, and molecular biology grade water (Sigma-Aldrich, St Louis, MO) 16 μl. The thermal cycling program was one cycle of 95°C for 3 min; 36 cycles of 95°C for 30 sec, 50°C for 45 sec, 72°C for 3 min; and final extension at 72°C for 7 min.

### TaKaRa Ex *Taq*™ DNA polymerase or combined with Dream*Taq*™

Each PCR amplification with TaKaRa Ex *Taq*™ DNA Polymerase (Takara Bio USA, Inc., Mountain View, CA) alone or combined with Dream*Taq*™ was carried out in a 25 μl of mixture containing TaKaRa Ex *Taq*™ (5 units/μl) 0.125 μl (or plus Dream*Taq*™ (5 units/μl) 0.125 μl), 10X Ex *Taq* Buffer 2.5 μl, dNTP (2.5 mM each) 2 μl, Template DNA 2 μl, forward primer 18S-CL-F3 (10 μm) 1.25 μl, reverse primer 28S-CL-R (10 μm) 1.25 μl, and molecular biology grade water (Sigma-Aldrich, St Louis, MO) 15.875 μl (or 15.75 μl). The thermal cycling program was: one cycle of 98°C for 30 sec; 36 cycles of 98°C for 10 sec, 68°C for 5 min; and final extension at 72°C for 7 min.

### PicoMaxx™ High Fidelity PCR System alone or combined with Dream*Taq*™

Each PCR amplification with PicoMaxx™ High Fidelity PCR System (PicoMaxx™ System) (Agilent, Santa Clara, CA) alone or combined with Dream*Taq*™ was carried out in a 25 μl of mixture containing PicoMaxx™ high fidelity PCR system (PicoMaxx™ (5 units/μl)) 0.5 μl (or plus Dream*Taq*™ (5 units/μl) 0.125 μl), 10× PicoMaxx™ reaction buffer (PicoMaxx™ buffer) 2.5 μl, dNTP (25 mM each) 0.2 μl, Template DNA 2 μl, forward primer 18S-CL-F3 (10 μm) 1.25 μl, reverse primer 28S-CL-R (10 μm) 1.25 μl, and molecular biology grade water (Sigma-Aldrich, St Louis, MO) 17.3 μl (or 17.175 μl). The thermal cycling program was one cycle of 95°C for 2 min; 36 cycles of 95°C for 30 sec, 55°C for 45 sec, 72°C for 5 min; and final extension at 72°C for 7 min.

### *pfu* DNA polymerase alone or combined with Dream*Taq*™:

Each PCR amplification with *pfu* DNA polymerase (Agilent, Santa Clara, CA) alone or combined with Dream*Taq*™ was carried out in a 25 μl of mixture containing *pfu* (2.5 units/μl) 0.75 μl (or plus Dream*Taq*™ (5 units/μl) 0.125 μl), 10× *Pfu* reaction buffer, 10× PicoMaxx™ buffer, or 10× Dream*Taq*™ buffer 2.5 μl, dNTP (25 mM each) 0.2 μl, Template DNA 2 μl, forward primer 18S-CL-F3 (10 μm) 1.25 μl, reverse primer 28S-CL-R (10 μm) 1.25 μl, and molecular biology grade water (Sigma-Aldrich, St Louis, MO) 16.85 μl (or 16.725 μl). The thermal cycling program was one cycle of 95°C for 2 min; 36 cycles of 95°C for 30 sec, 55°C for 45 sec, 72°C for 5 min; and final extension at 72°C for 7 min.

### *Pwo* DNA polymerase alone or combined with Dream*Taq*™

Each PCR amplification with *Pwo* DNA polymerase (Sigma-Aldrich, St Louis, MO) alone or combined with Dream*Taq*™ was carried out in a 25 μl final volume consisting of two mixtures: 12.5 μl of mixture A containing *Pwo* (5 units/μl) 0.125 μl (or plus Dream*Taq* (5 units/μl) 0.125 μl), 10× *Pwo* reaction buffer or 10× PicoMaxx™ buffer 2.5 μl, and molecular biology grade water (Sigma-Aldrich, St Louis, MO) 9.875 μl (or 9.75 μl); 12.5 μl of mixture B containing dNTP (25 mM each) 0.4 μl, template DNA 2 μl, forward primer 18S-CL-F3 (10 μm) 1.25 μl, reverse primer 28S-CL-R (10 μm) 1.25 μl. The thermal cycling program was one cycle of 95°C for 2 min; 36 cycles of 95°C for 30 sec, 57°C for 45 sec, 72°C for 5 min; and final extension at 72°C for 7 min.

### Herculase® II Fusion DNA polymerase

Each PCR amplification with Herculase® II Fusion DNA polymerase (Agilent, Santa Clara, CA) was carried out in a 25 μl of mixture containing Herculase® II Fusion DNA polymerase 0.5 μl, 5× reaction buffer 5 μl, dNTP (25 mM each) 0.25 μl, Template DNA 2 μl, forward primer 18S-CL-F3 (10 μm) 0.625 μl, reverse primer 28S-CL-R (10 μm) 0.625 μl, and molecular biology grade water (Sigma-Aldrich, St Louis, MO) 16 μl. The thermal cycling program was: one cycle of 95°C for 2 min; 36 cycles of 95°C for 20 sec, 55°C for 20 sec, 72°C for 2 min 15 sec; and final extension at 72°C for 7 min.

### Phusion™ High-Fidelity DNA polymerase

Each PCR amplification with Phusion™ High-Fidelity DNA Polymerase (Thermo Fisher Scientific, Waltham, MA) was carried out in a 25 μl of mixture containing Phusion™ High-Fidelity DNA Polymerase (2 units/μl) 0.25 μl, 5× reaction buffer 5 μl, dNTP (2.5 mM each) 0.5 μl, Template DNA 2 μl, forward primer 18S-CL-F3 (10 μm) 1.25 μl, reverse primer 28S-CL-R (10 μm) 1.25 μl, DMSO 0.25 μl, and molecular biology grade water (Sigma-Aldrich, St Louis, MO) 14.5 μl. The thermal cycling program was: one cycle of 95°C for 2 min; 36 cycles of 95°C for 20 sec, 55°C for 20 sec, 72°C for 2 min 15 sec; and final extension at 72°C for 7 min.

## Results

**Figure 1:**
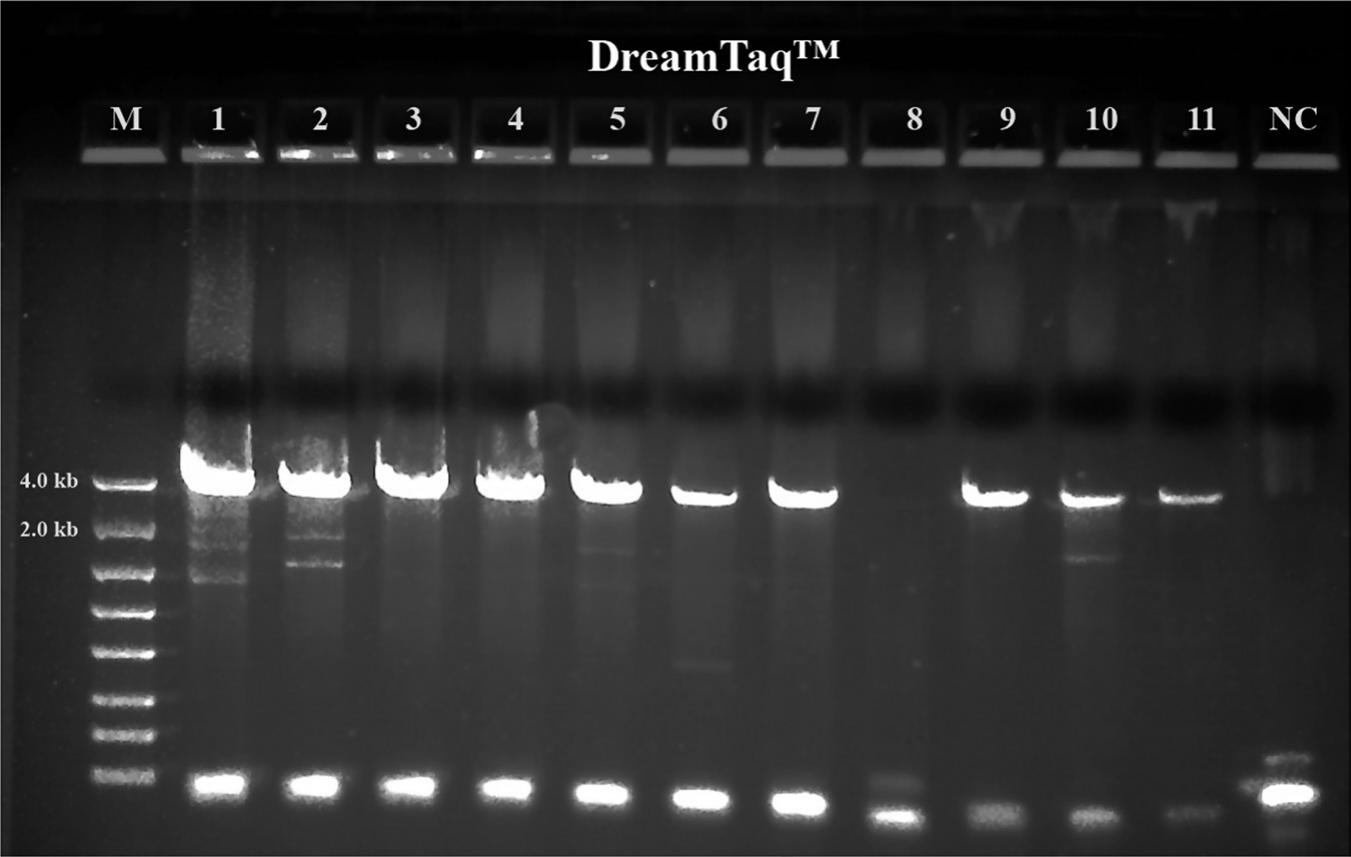
Long range ribosomal PCR Amplifications of the 3.5 kb target from Fall specimens with Dream*Taq*™. M: DNA markers; 1: 104H78; 2: 104H81; 3: 104H82; 4: 104H83; 5: 104H84; 6: 104H85; 7: 104H86; 8: 104H87; 9: 104H88; 10: 104H89; 11: 104H90; NC: negative control. 1-7: Female; 8-11: Male.

**Figure 2:**
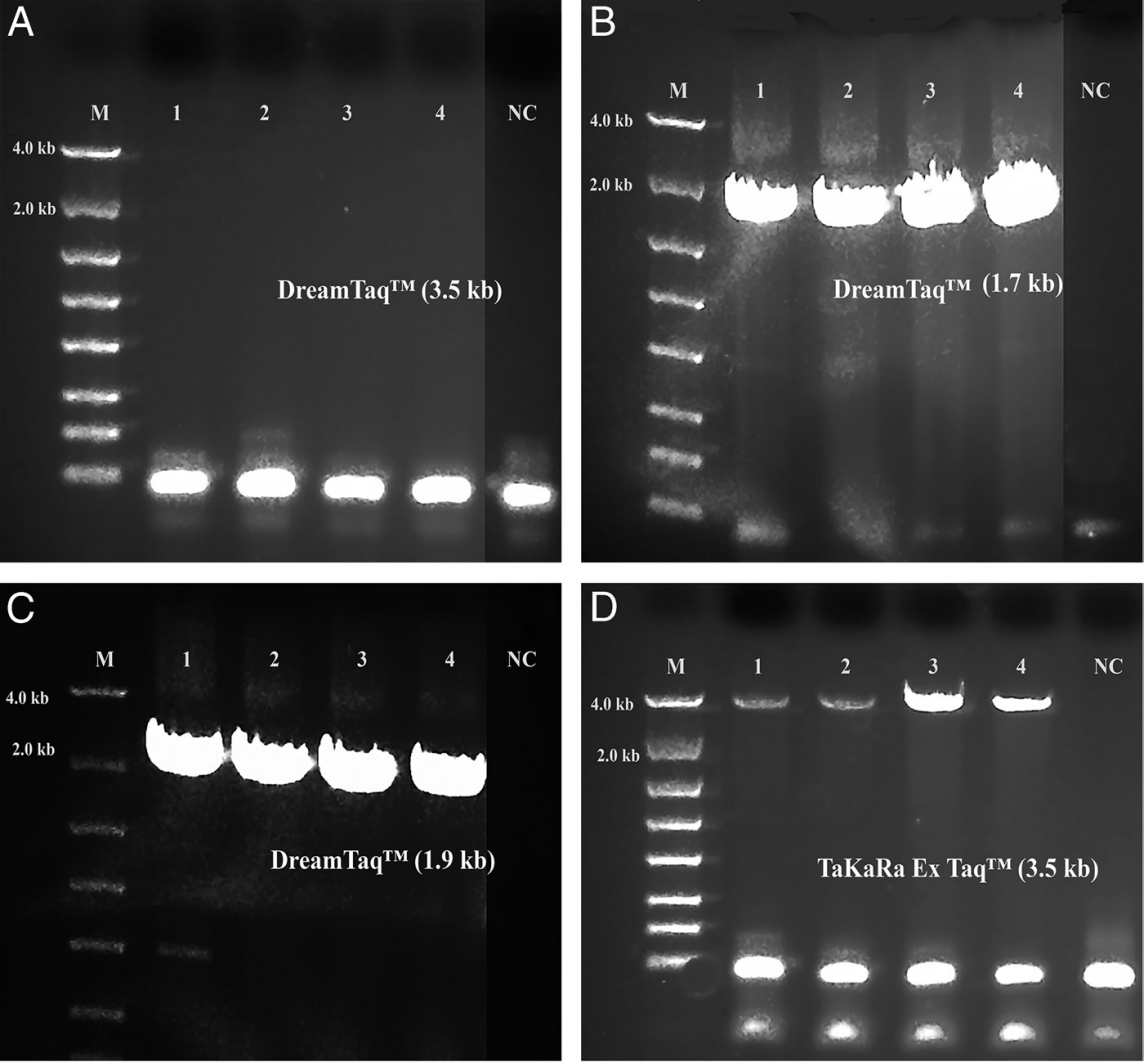
Long range ribosomal PCR Amplifications of the 3.5 kb target from Summer specimens with TaKaRa Ex *Taq*® system. M: DNA markers; 1: 104J54; 2: 104J55; 3: 104J58; 4: 104J59; NC: negative control, respectively. A: Dream*Taq*™; B: 18 S locus (1.7 kb) by Dream*Taq*™, C: ITS and 28 S loci (1.9 kb) by Dream*Taq*™; D: TaKaRa Ex *Taq*® system.

**Figure 3:**
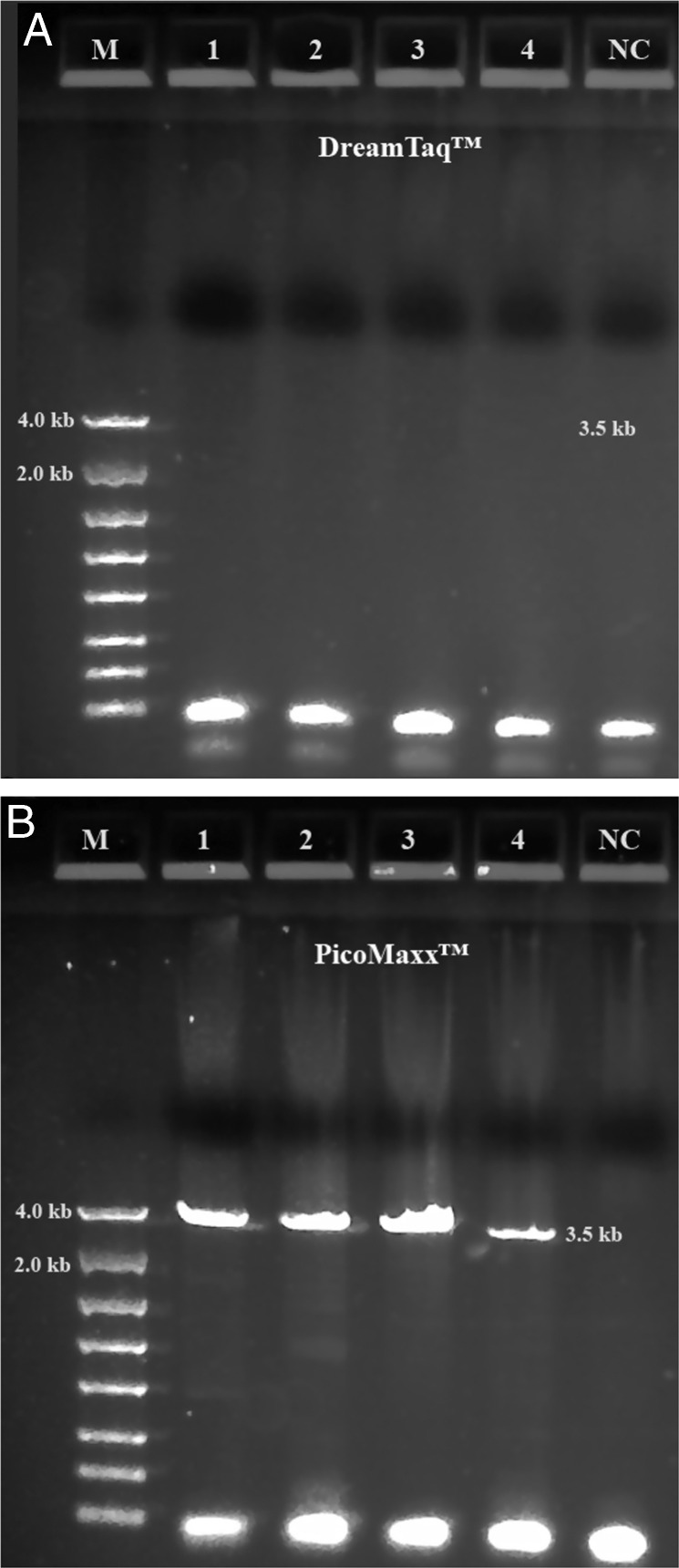
Long range ribosomal PCR Amplifications of the 3.5 kb target from Summer specimens with PicoMaxx™ High Fidelity PCR System. M: DNA markers; 1: 104K17; 2: 104K18; 3: 104K19; 4: 104K20; NC: negative control, respectively. A: Dream*Taq*™; B: PicoMaxx™ High Fidelity PCR System.

**Figure 4:**
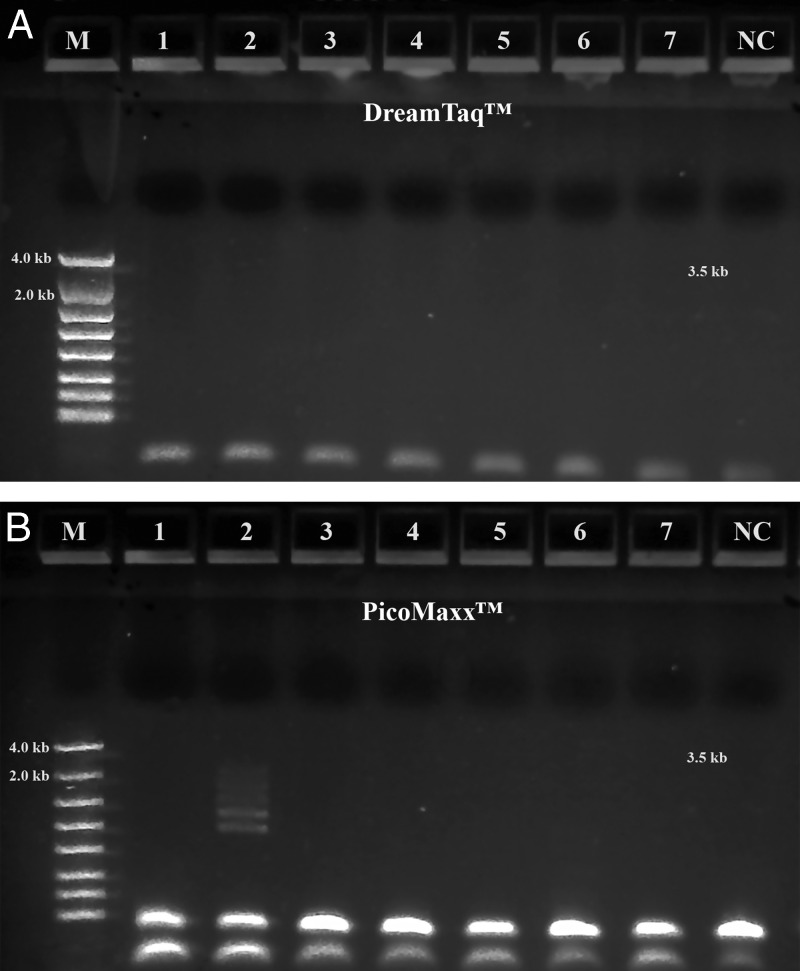
Long range ribosomal PCR Amplifications of the 3.5 kb target from Summer specimens with PicoMaxx™ High Fidelity PCR System. M: DNA markers; 1: 104K25; 2: 104K26; 3: 104K27; 4: 104K28; 5: 104K29; 6: 104K30; 7: 104K31; NC: negative control, respectively. A: Dream*Taq*™; B: PicoMaxx™ High Fidelity PCR System.

A summary of the PCR evaluations below based on specificity, efficiency and fidelity is given in Table 5 for individual polymerase systems, and in Table 6 for combined polymerase systems. Figure 1 shows that the successful 3.5 kb long segment PCR amplifications by the 18S-CL-F3 and 28S-CL-R primer set and the Dream*Taq*™ system were carried out in 10 out of 11 Fall specimens. The direct sequencing for the three loci (3.5 kb) was also conducted successfully in all specimens, except for 104H89 and 104H90 with low PCR yields that were good for sequencing only one or two loci. The 3.5 kb rDNA sequences generated for the specimens, 104H82 ( MN525396) and 104H83 (MN525397) were submitted to GenBank. This result shows that Dream*Taq*™ had the ability to amplify the 3.5 kb target in most Fall specimens within the size limit by *Taq* DNA polymerase up to 3 to 4 kb on amplicon (Erlich et al., 1991; Innis et al., 1988). However, failures (no yield for the 3.5 kb target) were observed in most Summer specimens (Fig. 2A, Fig. 3A, Fig. 4A). One possibility for this failure was amplicon size limitation associated with Dream*Taq*™ in these less mature Summer specimens. In order to address this issue, two medium segment PCR amplifications were carried out with Dream*Taq*™ and two primers sets, 18S-CL-F3/18S-CL-R7and ITS-CL-F2/28S-CL-R, which amplify the 18 S locus (1.7 kb), and ITS and 28 S loci (1.9 kb) within the 3.5 kb target, respectively. The amplifications showed that Dream*Taq*™ can amplify both medium 1.7 kb and 1.9 kb fragments with high yield (Fig. 2B, 2C), but not the 3.5 kb long targets (Fig. 2A) in these Summer specimens. This indicates that the amplification failure of the 3.5 kb long segment PCR in these Summer specimens is due to the size limitation of Dream*Taq*™ polymerase.

**Table 5.** Summary of PCR performance of Individual DNA polymerases (systems) tested in this study.

	Platinum™ *Taq*	*Taq*2000™	Dream*Taq*™	TaKaRa Ex *Taq*™	PicoMaxx™ System	*pfu*	*Pwo*	Herculase® II	Phusion™
Spring specimens	3.5 kb: X; 1.9 kb: √/X	3.5 kb: X; 1.9 kb: √√√√/X	3.5 kb: X; 1.9 kb: √√√√	na	na	na	na	na	na
Summer specimens	na	3.5 kb: X (DNS); 1.9 kb: √√√ (DNS)	3.5 kb: X or X/√ (DNS); 1.7Kb: √√√√/X; 1.9 kb: √√√√/X	3.5 kb: √√/X or X	3.5 kb: √√/X or X	3.5 kb: X (DNS)	3.5 kb: X (DNS)	3.5 kb: X	3.5 kb: X
Fall specimens	na	na	3.5 kb: √√√/X; 1.9 kb: NA	na	na	na	na	na	na

**Notes:** DNS, data not shown. The middle segments, 1.7 and 1.9 kb were not tested in all specimens, unless otherwise noted. X: An unsuccessful PCR amplification. It was defined practically as: weak or no target PCR bands (yields) on the Lonza gels. For example, weak target PCR bands shown Lane 1 and 2 in Figure 7B, which could not provide sufficient templates for downstream direct DNA sequencing. √: A successful PCR amplification. It was defined practically based on the observations from our routine direct DNA sequencing as: the amount of resulting target amplicon in the 25 μl of PCR reaction is good for at least 6 sequencing reactions (√) in downstream direct DNA sequencing, for example, the target PCR band shown on the Lonza gel on lane 11, in Figure 1 is good for at least 6 sequencing reactions (√), while each of the strong target PCR bands shown on the Lonza gel on lane 1 to 7 and 9 in Figure 1 is good for at least 18 sequencing reactions (√√√). /X: In a test or repeating tests, most PCR amplifications were successful and only one or few were unsuccessful. /√: In a test or repeating tests, most PCR amplifications were unsuccessful and only one or few were successful

**Figure 7:**
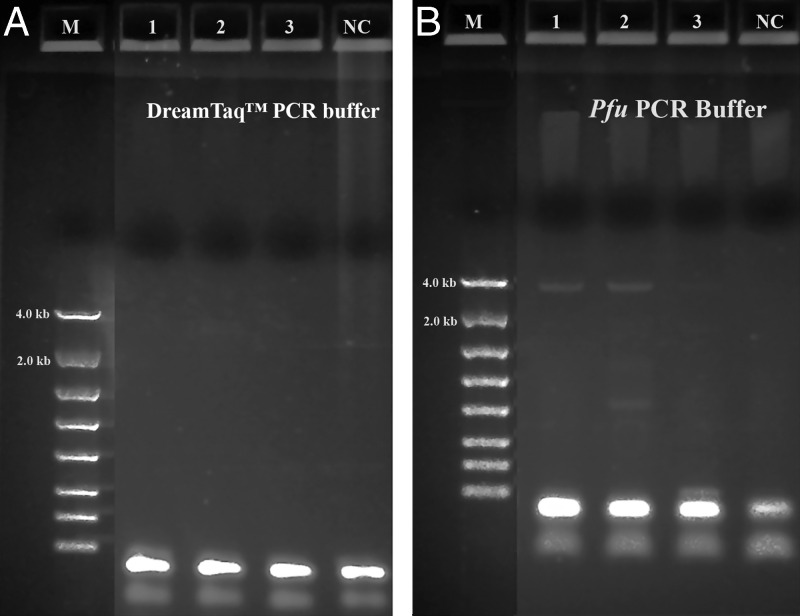
Long range ribosomal PCR Amplifications of the 3.5 kb target from Summer specimens with both Dream*Taq*™ and *Pfu* in manufacturer’s PCR buffers. M: DNA markers; 1: 104K29; 2: 104K30; 3: 104K31; NC: negative control, respectively. A: Dream*Taq*™ PCR buffer; B: *Pfu* PCR buffer.

**Table 6.** Summary of PCR performance of blended DNA polymerases (systems) tested in this study.

	TaKaRa Ex *Taq*™ combined with Dream*Taq*™ in TaKaRa Ex buffer	PicoMaxx™ System combined with Dream*Taq*™ in PicoMaxx™ buffer	Dream*Taq*™ in PicoMaxx™ buffer	*pfu* in PicoMaxx™ buffer	*Pfu* combined with Dream*Taq*™ in PicoMaxx™ buffer	*pfu* combined with Dream*Taq*™ in Dream*Taq*™ buffer	*pfu* combined with Dream*Taq*™ in *pfu* buffer	*Pwo* in PicoMaxx™ buffer	*Pwo* combined with Dream*Taq*™ in *Pwo* buffer	*Pwo* combined with Dream*Taq*™ in PicoMaxx buffer
Spring specimens	na	na	na	na	na	na	na	na	na	na
Summer specimens	3.5 kb: X	3.5 kb: √√√/X	3.5 kb: √/X or X	3.5 kb: X	3.5 kb: √√√√ or √√	3.5 kb: X	3.5 kb: X	3.5 kb: X	3.5 kb: X (DNS)	3.5 kb: √/X
Fall specimens	na	na	na	na	na	na	na	na	na	na

**Note:** See the notes in Table 5.

**Figure 5:**
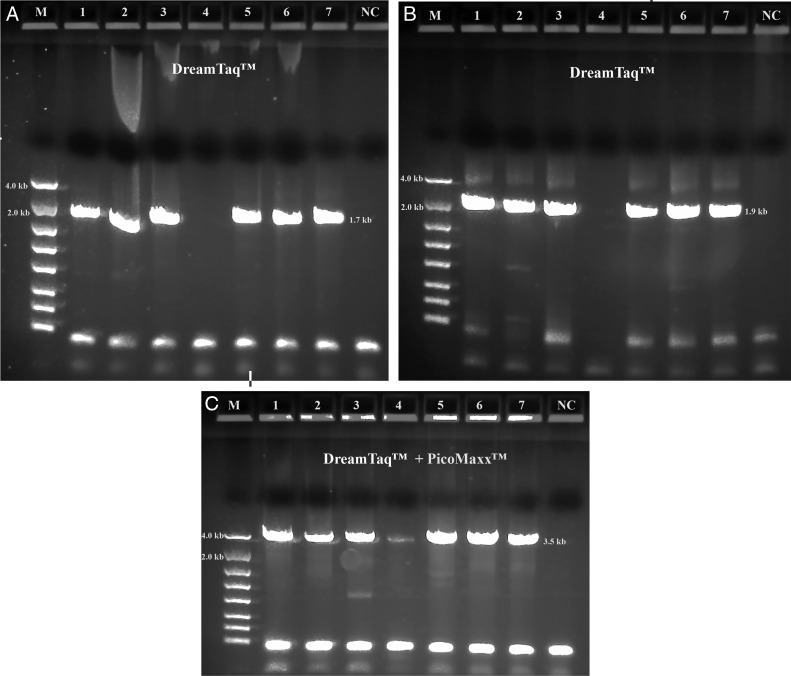
Long range ribosomal PCR Amplifications of the 3.5 kb target from Summer specimens with Dream*Taq*™ and PicoMaxx™ High Fidelity PCR System. M: DNA markers; 1: 104K25; 2: 104K26; 3: 104K27; 4: 104K28; 5: 104K29; 6: 104K30; 7: 104K31; NC: negative control, respectively. A: 18 S locus (1.7 kb) by Dream*Taq*™, B: ITS and 28 S loci (1.9 kb) by Dream*Taq*™; C: Dream*Taq*™ and PicoMaxx™ High Fidelity PCR System combined.

**Figure 6:**
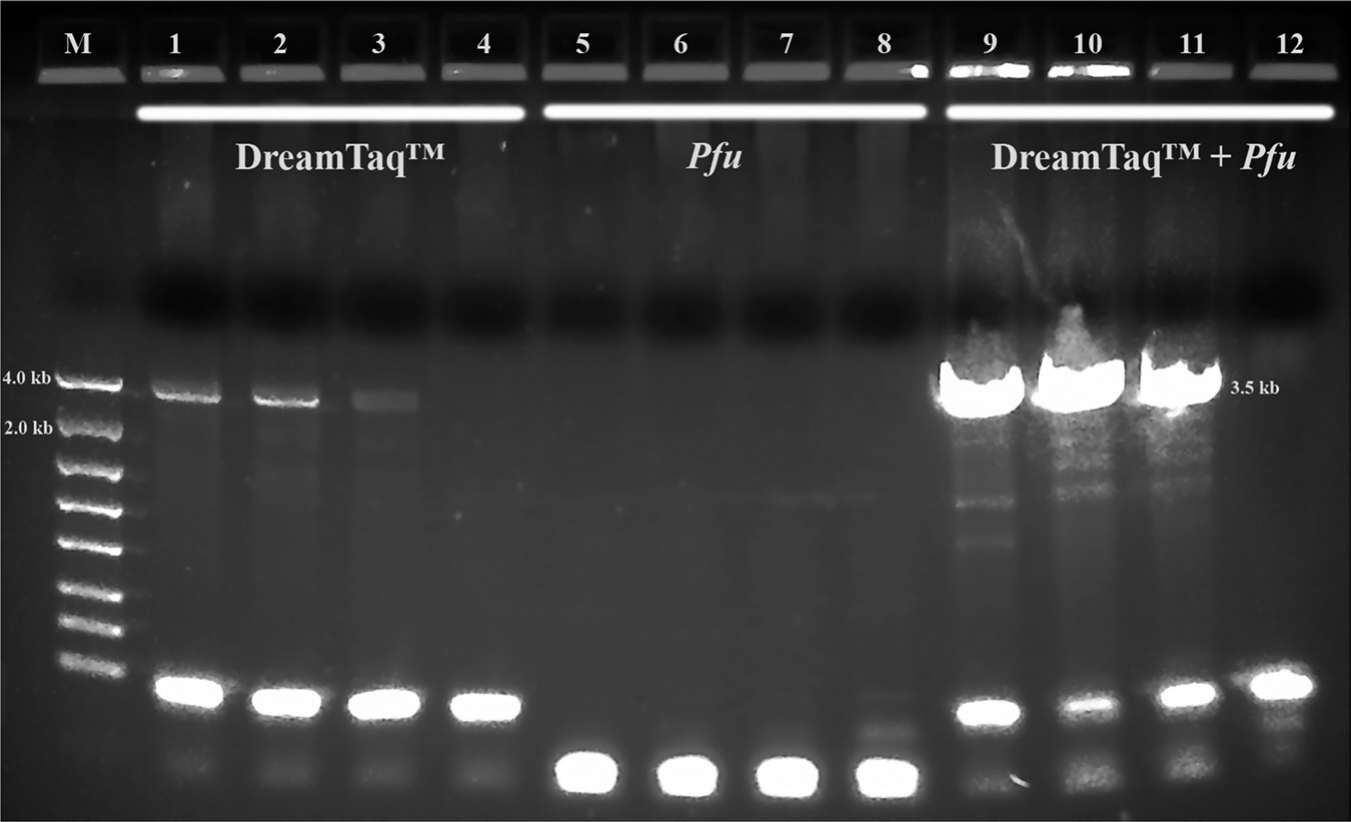
Long range ribosomal PCR Amplifications of the 3.5 kb target from Summer specimens with Dream*Taq*™ or/and *Pfu* in PicoMaxx™ buffer. M: DNA markers; 1, 2, 3 and 4: Dream*Taq*™; 5, 6, 7 and 8: *Pfu*; 9, 10, 11and 12: Dream*Taq*™ and *Pfu* combined; 1, 5 and 9: 104K29; 2, 6 and 10: 104K30; 3, 7 and 11: 104K31; 4, 8 and 12: negative control (NC), respectively.

**Figure 9:**
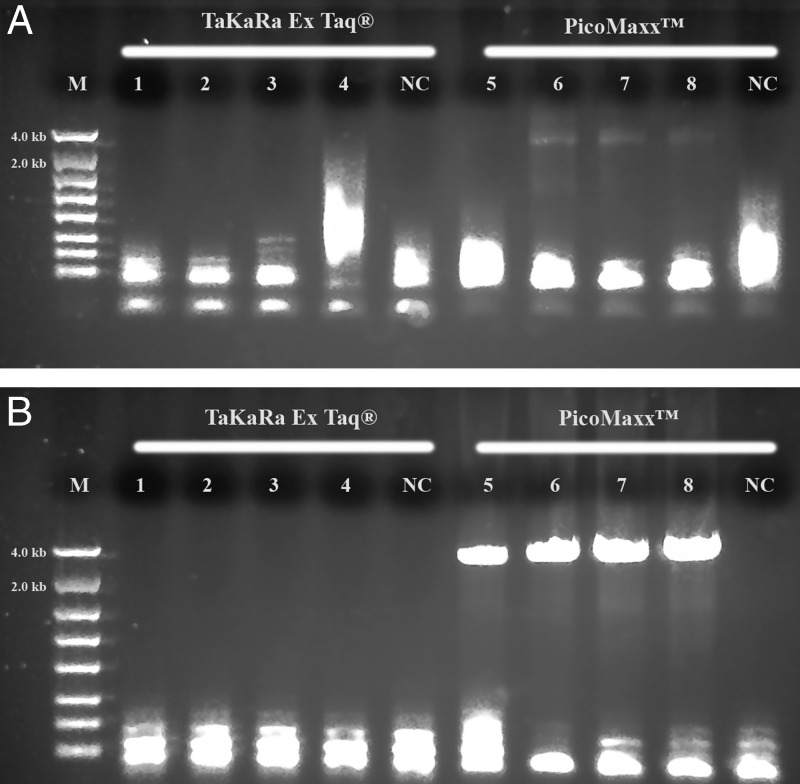
PCR performance of TaKaRa Ex *Taq*® system and PicoMaxx™ High Fidelity PCR System. M: DNA markers; 1 and 5: 104K37; 2 and 6: 104K38; 3 and 7: 104K39; 4 and 8: 104K40. A: 1, 2, 3 and 4: TaKaRa Ex *Taq*® system; 5, 6, 7 and 8: PicoMaxx™ High Fidelity PCR System; B: 1, 2, 3 and 4: TaKaRa Ex *Taq*® system and Dream*Taq*™; 5, 6, 7 and 8: PicoMaxx™ High Fidelity PCR System. NC: negative control, respectively.

Both TaKaRa *Taq*® and PicoMaxx™ systems can amplify the 3.5 kb target in some of the Summer specimens in which the Dream*Taq*™ failed (compare Fig. 2A with 2D and Fig. 3A with 3B). However, they also failed to amplify the 3.5 kb target in other Summer specimens (Fig. 4B; Fig. 9A (Lanes 5, 6, 7)). In order to establish a system to amplify the 3.5 kb target regardless of the variations of specimens, Dream*Taq*™ and PicoMaxx™ were combined to test if both work together to overcome this difficulty during the long segment PCR. In Figure 5A, 5B, Dream*Taq*™ demonstrated again its ability to amplify both 1.7 kb and 1.9 kb medium segment fragments within the 3.5 kb target in the difficult specimens. Figure 5C shows the robust amplifications of the 3.5 kb target for these specimens by the combination of Dream*Taq*™ and the PicoMaxx™ System, which suggests it is the proofreading ability by *Pfu* in PicoMaxx™ that greatly facilitates Dream*Taq*™. In order to elucidate the synergy between the two, reconstituting Dream*Taq*™ and the *Pfu* used in the PicoMaxx™ System was conducted. In the presence of the PicoMaxx™ buffer for these difficult specimens (Fig. 6), the Dream*Taq*™ alone barely amplified the 3.5 kb target (Lanes 1, 2, 3, Fig. 6). The proofreading *Pfu* by itself failed to amplify the product (Lanes 5, 6, 7, Fig. 6), but combining the Dream*Taq*™ and the *Pfu* delivered a robust amplification (Lanes 9, 10, 11, Fig. 6). We tested whether the combination works in the presence of either Dream*Taq*™ buffer or *Pfu* buffer. However, neither of them could facilitate the combination (Fig. 7A, 7B). This suggests that a long segment PCR may not be achieved by simply blending a *Taq* with a proofreading DNA polymerase, but the PCR buffer must be taken into account as well. In this system (Lanes 9, 10, 11, Fig. 6), the three proprietary components, Dream*Taq*™, *Pfu* and PicoMaxx™ buffer must be purchased separately, which is not economical. Therefore, the combination of Dream*Taq*™ and PicoMaxx™ High Fidelity PCR System (*Taq, Pfu* and buffer) seen in Figure 5C is the preferable option to address difficult specimens.

**Figure 8:**
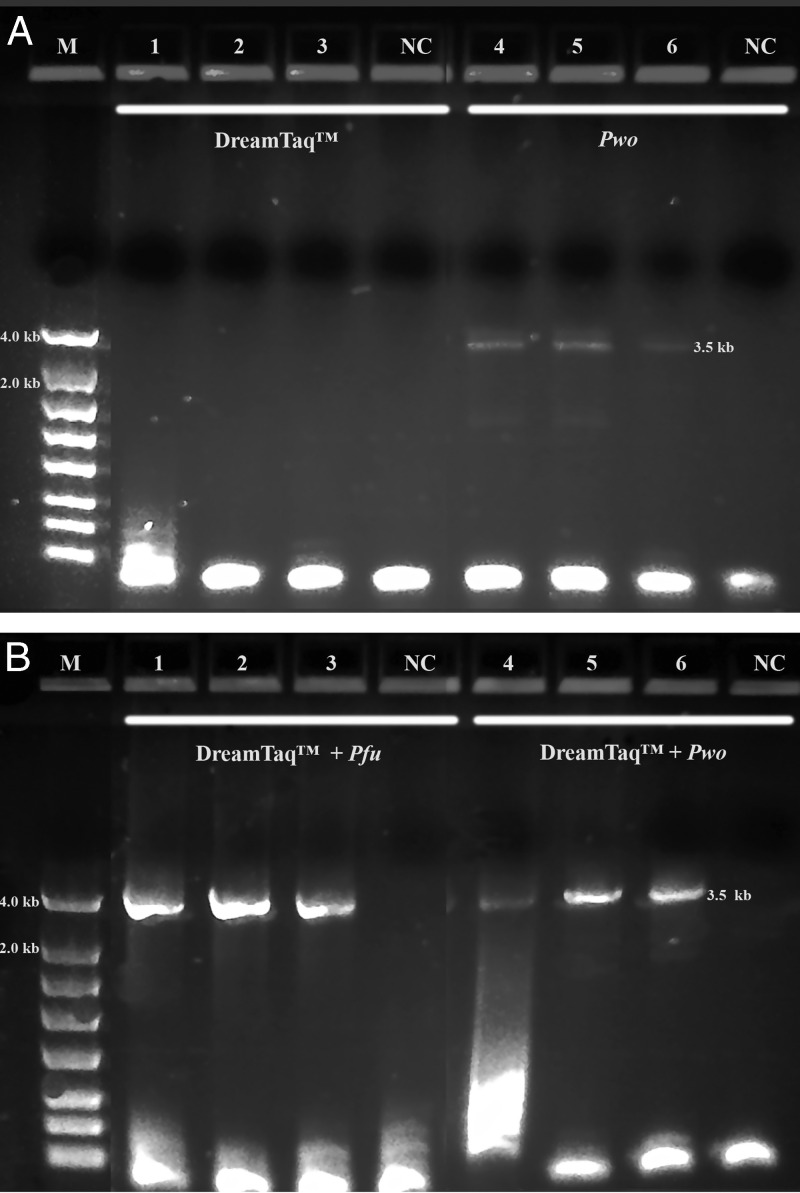
PCR performance of *Pfu* and *Pwo* in PicoMaxx™ buffer. M: DNA markers; 1 and 4: 104K37; 2 and 5: 104K38; 3 and 6: 104K39. A: 1, 2 and 3: Dream*Taq*™; 4, 5 and 6: *Pwo* (0.125 μl per reaction). B: 1, 2 and 3: Dream*Taq*™ and *Pfu*; 4, 5 and 6: Dream*Taq*™ and *Pwo* (0.125 μl per reaction). NC: negative control, respectively. Note: final concentration of *Pfu* in each reaction was aligned with *Pwo* and Dream*Taq*™ in 0.625 units.

*Pwo* (derived from *Pyrococcus woesei*), another proofreading DNA polymerase, was tested in line with the *Pfu* in PicoMaxx™ buffer. Figure 8 shows that in the presence of the PicoMaxx™ buffer, both combinations (Lanes 1, 2, 3, 4, 5, 6 in Fig. 8B) are better than either *Pwo* or Dream*Taq*™ alone (Lanes 1, 2, 3, 4, 5, 6 in Fig. 8A), and the combination with *Pfu* performed better than the combination with *Pwo* (Fig. 8B). The presence of either Dream*Taq*™ buffer or *Pwo* buffer was also evaluated for the combination of Dream*Taq*™ and *Pwo* in a different specimen from beech buds collected in the spring of 2019 (Spring specimens). No significant amplifications of the 3.5 kb target were seen in the presence of either buffer (data not shown). This confirms again that PCR buffer is another key to the success of the Dream*Taq*™ and *Pfu* or *Pwo* combination.

The comparison between TaKaRa Ex *Taq*® system and PicoMaxx™ system was also performed. Figure 9A shows that both PicoMaxx and TaKaRa Ex *Taq*® systems failed to amplify the 3.5 kb target, but the PicoMaxx™ system gave Dream*Taq*™ dramatic leverage over the TaKaRa Ex *Taq*® system (Fig. 9B), and again the combination of Dream*Taq*™ and the PicoMaxx™ System demonstrated its robust long segment PCR amplification in the difficult specimens.

**Figure 10:**
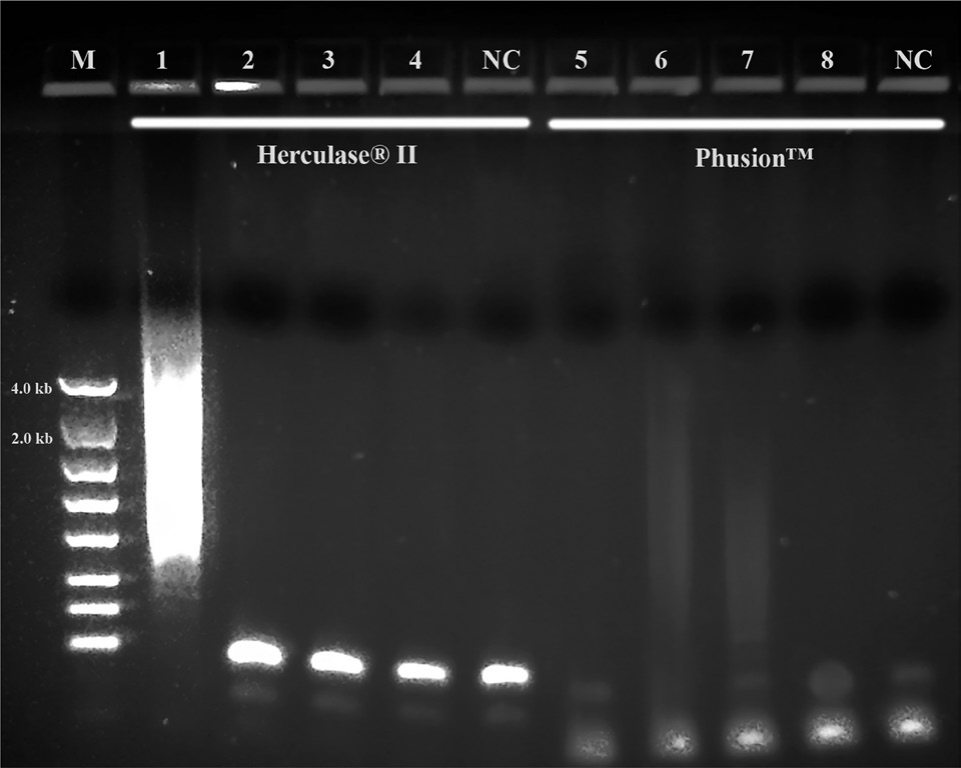
PCR performance of Herculase® II Fusion DNA polymerase and Phusion™ High-Fidelity DNA Polymerase. M: DNA markers; 1 and 5: 104K37; 2 and 6: 104K38; 3 and 7: 104K39; 4 and 8: 104K40. 1, 2, 3 and 4: Herculase® II Fusion DNA polymerase; 5, 6, 7 and 8: Phusion™ High Fidelity PCR System; NC: negative control, respectively.

Fusion DNA polymerase is an engineered fusion of a proofreading polymerase and a processivity-enhancing domain (Ishino and Ishino, 2014) and offers tremendous advantages over traditional *Taq* with high fidelity, robust amplification in low abundance, high GC, and other difficult targets, short extension times (1.0 kb/10-15 sec) and ability to amplify long target (>20 kb) (both Agilent and Thermo Fisher Scientific web sites). Herculase® II Fusion DNA polymerase and Phusion™ High-Fidelity DNA Polymerase were tested. Figure 10 shows both could not produce any 3.5 kb target bands except for the smear band by the Herculase® II Fusion DNA polymerase.

**Figure 11:**
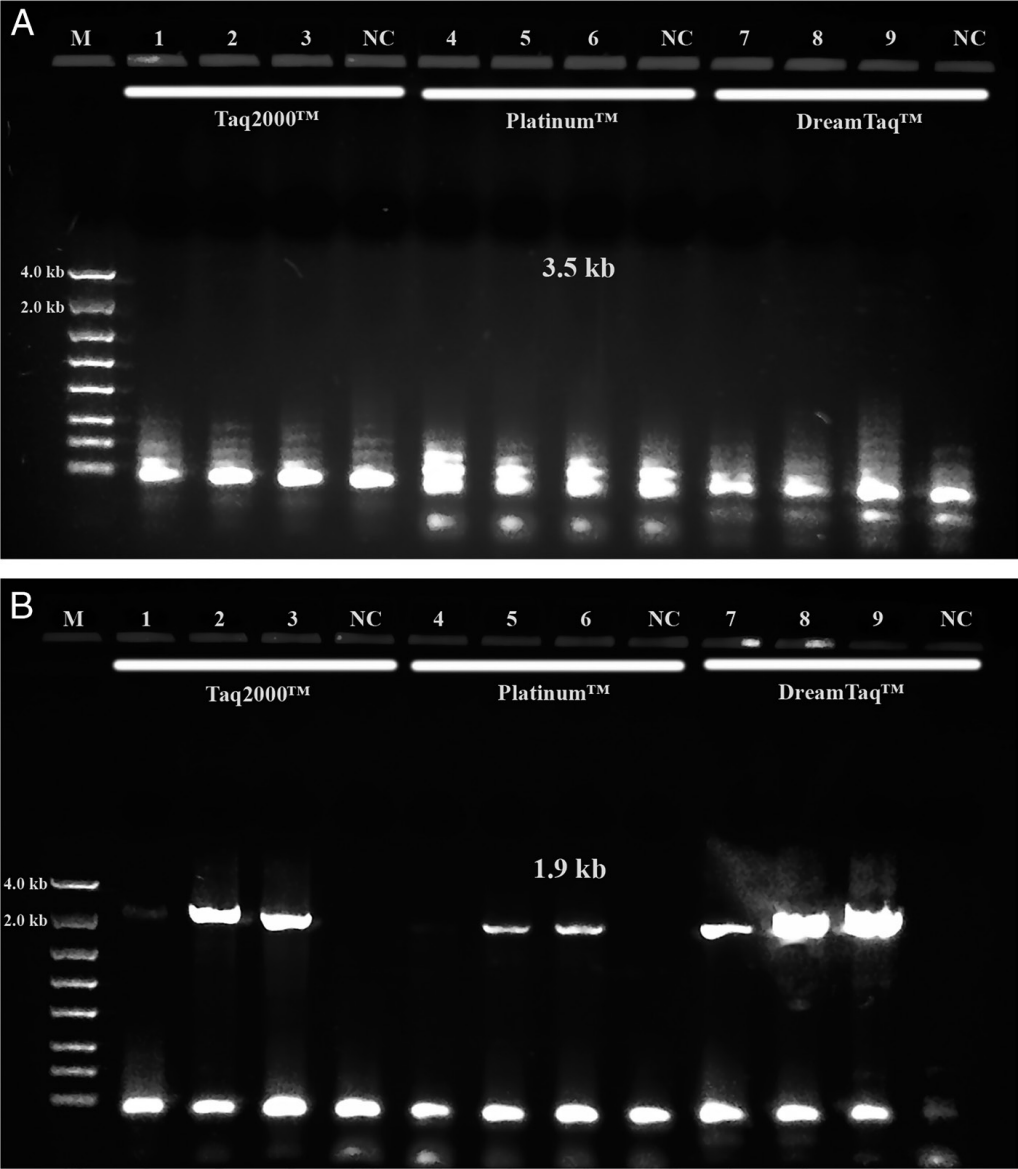
PCR performance of *Taq*2000™, Platinum™ *Taq* and Dream*Taq*™. M: DNA markers; 1, 4 and 7: 104N95; 2, 5 and 8: 104N96; 3, 6 and 9: 104N97. 1, 2, 3 and NC by *Taq*2000™; 4, 5, 6 and NC by Platinum™ *Taq*; 7, 8, 9 and NC by Dream*Taq*™, NC: negative control, respectively. A: 3.5 kb target; B: 1.9 kb ITS and 28 S target. Note: final concentration of either *Taq*2000™ or Dream*Taq*™ in each reaction was aligned with Platinum™ *Taq* in 1.25 units.

The PCR performances of *Taq*2000™, which is one of the components of the PicoMaxx™ system, Platinum™ *Taq* and Dream*Taq*™ were also compared in Spring specimens. In the presence of their own buffers, both the long segment PCR for the 3.5 kb target and the medium range PCR for the 1.9 kb target were carried out. In the long segment PCR amplifications, all of the three *Taqs* failed to amplify the 3.5 kb target (Fig. 11A). In the medium segment PCR amplifications, the Platinum™ *Taq* weakly amplified the 1.9 kb target (Lanes 5, 6 in Fig. 11B), the *Taq*2000™ was able to amplify two specimens successfully (Lanes 2, 3 in Fig. 11B), and the Dream*Taq*™ outperformed either the Platinum™ *Taq* or *Taq*2000™ in all three specimens (Lanes 7, 8, 9 in Fig. 11B). This indicates that Dream*Taq*™ has better sensitivity in this situation than either the Platinum™ *Taq* or *Taq*2000™. It also further explains why combining the PicoMaxx™ system (PicoMaxx™ and PicoMaxx™ buffer) and Dream*Taq*™ can successfully amplify the 3.5 kb target in the specimens where both Dream*Taq*™ and PicoMaxx™ systems failed separately.

The 3.5 kb targets amplified by the *Taq*-based blend systems, TaKaRa Ex *Taq*® DNA Polymerase in the specimen 104J58 (OH), PicoMaxx™ High Fidelity PCR System in the Summer Specimen 104K17 (OH); by the combination of Dream*Taq*™ and PicoMaxx™ High Fidelity PCR System in the Summer specimens, 104K25 (Potter County, PA) and 104K37 (Crawford County, PA) were sequenced and the resulting rDNA sequences (ITS and 28 S loci) were deposited in GenBank with the accession numbers, 104H82, MN525396; 104H83, MN525397; 104J58 MN525398; 104K17, MN525399; 104k25, MN525400; 104K37, MN525401, respectively. Multiple alignments of these sequences above with the 3.5 kb rDNA (MK292137 and MK292138) of the Ohio *Litylenchus* specimens in the previous study (Carta and Li, 2019) reveal that the ITS and 28 S (D1D2D3) sequences of the Pennsylvania specimens are 100% identical to the Ohio specimens.

## Discussion

The molecular taxonomic identifications performed in this study not only confirmed that the nematodes discovered in BLD leaves from Ohio and Pennsylvanian are the same species of *Litylenchus crenatae mccannii*, but they also demonstrated a technical improvement to achieve consistent amplification of the 3.5 kb ribosomal PCR product through long segment PCR amplification using sometimes variable quality crude genomic DNA extracts as template.

We observed that most Fall specimens were mature and not very active, but with highly developed reproductive systems containing many germ cells. In contrast, most Summer specimens were young, motile adults with a poorly developed reproductive system. The cuticle is the first barrier for proteinase K to lyse in a nematode which is relatively tough and resistant to environmental forces in young adults, but loose, wrinkled, permeable and susceptible to environmental insults in older adults (Searcy et al., 1976; Davies and Curtis, 2011; Herndon et al., 2017). These differences mean the Fall Specimen nematode cuticles are more easily broken down by proteinase K to release more genomic DNA. This is especially favorable for long DNA fragments (i.e. larger than 3.5 kb) which have more unlysed debris and impurities than fragments found in younger Summer specimens. Thus, both the quantity of the 3.5 kb target template and the debris and impurities in the crude unpurified genomic DNA lysates may vary from session to session and specimen to specimen. This phenomenon was demonstrated by the successful amplification of the 3.5 kb target by Dream*Taq*™ in Fall specimens (Fig. 1), but not in the Summer specimens (Figs. 1A and 2A). The debris or impurities in the incomplete genomic DNA lysates from Summer specimens may interfere with the PCR extension of the 3.5 kb target by increasing the probability of incorporating wrong nucleotides and consequently increasing the size limitation associated with Dream*Taq*™ and eventually failing the 3.5 kb long segment PCR, but not the medium segment PCR amplification (Fig. 2A, 2B, 2C). This failure can be prevented by a proofreading DNA polymerase (either TaKaRa Ex *Taq*® system or the PicoMaxx™ system) in these Summer specimens (Figs. 2D and 3B).

When both Dream*Taq*™ and these *Taq*-based blend systems failed (Fig. 4), both the low quantity of the 3.5 kb long fragments and the debris and impurities in the input DNA were the apparent causes. These double failures were prevented by employing both Dream*Taq*™ and the PicoMaxx™ system (Fig. 5C). Per the vendor’s information, Dream*Taq*™ DNA Polymerase could amplify a target from as low as 3 pg of purified human genomic DNA, and provided higher sensitivity compared to six other *Taq* DNA polymerases, including TaKaRa *Taq*® DNA Polymerase (a component of the TaKaRa Ex *Taq*® system). Dream*Taq*™ also demonstrated its higher sensitivity than either *Taq*2000™ or Platinum™ *Taq* (Fig. 11B). The failures of the PicoMaxx™ system were caused by the low sensitivity of its *Taq*2000™ DNA polymerase and were reversed by adding Dream*Taq*™ (Figs. 5C and 9B) because of its high sensitivity. The direct synergy between Dream*Taq*™ and proofreading *Pfu*, the key component in the PicoMaxx™ system, is further confirmed in Figure 6. In the combination of Dream*Taq*™ and the PicoMaxx™ system, Dream*Taq*™ and proofreading *Pfu* worked synergistically only in the presence of PicoMaxx™ buffer (Fig. 6 Lanes 9, 10, 11, Fig. 7A, 7B). Both *Pfu* and *Pwo* were also compared directly in this study (Fig. 8). The synergy with Dream*Taq*™ was not supported by either of their own buffers, but PicoMaxx™ buffer allowed *Pfu* to perform more robustly than *Pwo*. Therefore, the PCR buffer is also required for successful synergy between Dream*Taq*™ and *Pfu* or *Pwo*. Tests in two Fusion DNA polymerases suggest Fusion DNA polymerases may not be suitable for crude unpurified genomic DNA in low quantity and quality although they have multiple advantages over traditional *Taq* (Fig. 10).

Taken together, the size limit to the 3.5 kb target by *Taq*, the low quantity of the 3.5 kb target template and the debris and impurities in the crude and unpurified genomic DNA lysates are three primary factors responsible for the failures of the 3.5 kb long PCR amplification in the Summer specimens. Establishing the combination of Dream*Taq*™ and the PicoMaxx™ system in this study well addressed the issues above for the 3.5 kb long segment ribosomal PCR amplification by combining the sensitivity of Dream*Taq*™, the proofreading of *Pfu* and the sensitivity and robustness of PicoMaxx™ buffer. In this study long segment ribosomal PCR amplification in various *Litylenchus* specimens has been achieved by this technical improvement. Successful long ribosomal PCR by this improvement was also conducted for other taxa, *Ditylenchus* sp. (Tylenchida), *Pristionchus* sp. (Rhabditida) and *Prodorylaimus* sp. (Dorylaimida) when their specimens were difficult to amplify with the one primer set and Dream*Taq*™ (data not shown). This improvement provides high fidelity, sensitivity and yield with minimum optimization of reaction and cycling conditions. It should not be limited to long segment PCR amplification only, and could be considered for short range PCR with forensic or ancient DNA, single copy nuclear gene PCR or where improved proofreading can rescue mismatches that take place between the 3’ primer termini and its target templates.
